# The Secrets of Alexis

**Published:** 1894-07-14

**Authors:** 


### The First Century of English Medical Literature.

### VII.—THE SECRETS OF ALEXIS.

Few books attained to greater household popularity in the latter half of the 16th century than this notable collection of Don Alexis, done into English by William Warde. Several editions appeared in quick succession in the course of a few years, and Bayle informs us that "the hawkers carry them to country fairs, with their other little books, covered with blue paper." Even in those days there was no surer test of popular favour than being sold in a cheap edition.

Nothing is known of Don Alexis beyond what he chooses to reveal in his preface, and his account of himself is so delicately tinted with rose, and the truths for which he solemnly vouches elsewhere, are of so startling a nature, that the reader must please himself whether to believe all or none. Briefly he gives himself out to have passed a long life in study, and to be learned in the "Latin, Greeke, Hebrew, Caldei, and Arabick tong," not to mention those of divers other nations and countries; now in extreme old age he sets out to give his jealously guarded secrets to the world, moved by remorse at the death of an artificer in Milan, whom a timely disclosure of his methods to the surgeon in charge might have cured. One wise caution he adds to his reader, that "with medicines concerning man's body he uses the ayde and help of physitions, although, indeed, many of them, with a passion of jealousy, are wont to blame and to contempne things that come not of themselves." Lastly, if some of the prescriptions have not the desired effect, the patient is to "beginne again with more diligence, assuring himself that there is nothing in this book but is true and experimented."

Upon this we pass to the first "secret," which is neither more nor less than "the manner and secrete to conserve a man's youth, and to holde back old age," attested by actual experiment on "an old man of three score and ten years, all withered with age, of a very evill complexion, and subject to divers kinds of diseases, who was altered and changed as unto the age of six or eight and twenty years." No reasonable man would expect this sort of thing to be done in a minute; accordingly, you set about it in a leisurely way, collecting dew on fine May mornings that is fallen upon "rosemarie, burrage, and other good herbs (sage only excepted), for that under Sage certain venomous beasts are wont to assemble, which infect and poyson it with their breath." A good many little things have next to be got together, as sugar, manna, honey, quinces, agario, roses, fumitory, hops, March violets, and aloes. An ounce of small fine pearls is an important item, dissolved in lemon juice and added to a like quantity of red coral, mixed with blue vitriol, and well burned in a close pot. The mixture must next be made "slab and good" by processes too numerous to mention, and a spoonful may then be taken two or three times a week "in half a glassful of the milk of a woman, new brought to bed of a man childe." The whole secret is redolent of the perfume of some old-fashioned garden, and might well make a man young again merely in the preparation.

The same cannot be said of a recipe for making "an oyle of a red dog, by the meane whereof I have healed a Frier of S. Onofres, who had by the space of twelve yeares a lame and drie withered arm like a stick." There are who would find the infirmity more tolerable than association in the following process: You take a young dog of red hair and you keep him three days without meat. Then you mercifully strangle him with a cord. Meantime you boil a kettle of oil on the fire, and put your dog in, whole or in pieces, no matter how, so that he be all there with the skin and the hair. You are now to take scorpions to the number of four score or a hundred, and go through much the same process, omitting the strangling. You add a few more creeping things which need not be specified, a handful of S. John's Wort, three tortoises (if you can get them) that live on the land and not in the water, seven ounces of the "gambon" of a hog, and, to complete all, a pound of flesh from the hinder thighs of an ass. It has "infinite virtues," and is a sure cure for the gout.

Not all the secrets are of this ambitious character. A very large proportion deal with what a writer of the period denominates "the unsearchable deceits of women," and read more like the answers to correspondents in a modern ladies' newspaper than the prescriptions of a reverend physician. Of face washes there are great plenty, and of the craftiest description. "A very good water to make the face appear of the age of five and twentie yeeres," is distinctly outdone a little further on by "another water to beautifie the face and to make it appear of the age of fiftene years." Then we have the manner "to make a very excellent red colour for the face, which is natural and continueth long upon the face, making it always gayer and fairer," and there is "a goodly way or manner how to make yellow aberne hair," besides countless other hair washes, and recipes for removing "superfluous hairs," as the phrase now goes.

"For to see wild beasts in a dream," for which there are two prescriptions, is a secret most people could go wanting. To make "that one have a good memory," or "that no dog shall bark at you," is more tempting, and these little things are very easily managed if you can get hold of the eye of a black dog or the tooth of a badger and know what to do with them. It is the making of all the pomades, tooth-powders, soaps, and perfumes which must have strained the household resources, so complicated are the processes and so innumerable the ingredients. The contemplation of these things might go far to reconcile a man even to some aggressive advertising on the part of the purveyors who have banished the necessity for home-made cosmetics.

Perhaps the quaintest secret is "to make that a woman shall eate of nothing that is sette upon the table." "Take a little greene Basil, and when men bring the dishes to the table, put it underneath them that the woman perceive it not; for men say that she will eat of none of that which is in the dish whereunder the Basil lyeth." Has any one ever tried this?

